# 

**Published:** 1886-11

**Authors:** 


10 cts. a copy. NOVEMBER, 1886. $1.00 per year

### HALL'S JOURNAL OF HEALTH

ESTABLISHED 1854

OFFICE: 206 BROADWAY, EVENING POST BUILDING, Room 11. N. Y.

Entered as Second-class Matter at the Post-Office, New York.

